# Measurement of the “Safe Zone” and the “Dangerous Zone” for the Screw Placement on the Quadrilateral Surface in the Treatment of Pelvic and Acetabular Fractures with Stoppa Approach by Computational 3D Technology

**DOI:** 10.1155/2014/386950

**Published:** 2014-01-29

**Authors:** Sheng Zhang, Wanhan Su, Qiang Luo, Frankie Leung, Bin Chen

**Affiliations:** ^1^Department of Orthopedics & Traumatology, Nanfang Hospital, Southern Medical University, No. 1838 North Guangzhou Avenue, Guangzhou 510515, China; ^2^Department of Orthopedics & Traumatology, Queen Marry Hospital, The University of Hong Kong, Pok Fu Lam, Hong Kong

## Abstract

This study is aimed at definition of the safe and dangerous zone for screw placement with Stoppa approach for rapid identification during operation and a new way for the studies on the “safe zone.” Pelvic CT data of 84 human subjects were recruited to reconstruct the three-dimensional (3D) models. The distances between the edges of the “safe zone,” “dangerous zone,” and specific anatomic landmarks such as the obturator canal and the pelvic brim were precisely measured, respectively. The results show that the absolute “dangerous zone” was from the pelvic brim to 3.07 cm below it and within 2.86 cm of the obturator canal, while the region 3.56 cm below the pelvic brim or 3.85 cm away from the obturator canal was the absolute “safe zone” for screw placement. The region between the absolute “safe zone” and the absolute “dangerous zone” was the relatively “dangerous zone.” As a conclusion, application of computer-assisted 3D modeling techniques aids in the precise measurement of “safe zone” and “dangerous zone” in combination with Stoppa incision. It was not recommended to place screws on the absolute dangerous zone, while, for the relatively “dangerous zone,” it depends on the individual variations in bony anatomy and the fracture type.

## 1. Introduction

Surgery and secured internal fixation are required for the majority of pelvic and acetabular fractures with displacement to achieve an anatomical reduction and a good prognosis [1]. Currently, the anterior ilioinguinal approach reported by Matta [2] and stöckle et al. [3] is most commonly used in treating patients with pelvic and acetabular fractures. Due to the fact that three exposure windows are required for this procedure, it may increase the difficulty of the surgery, and inner fixation of screws on the quadrilateral surface is also difficult. With the Stoppa approach, placement of screws or plates on the quadrilateral surface has become possible [4]. Previous studies had well described the surgical exposures and operation technique. And good outcomes using the modified Stoppa approach for treating pelvic and acetabular fractures were obtained, which was first reported by Cole and Bolhofner [5] and was followed by Ponsen et al. [6], Andersen et al. [7], and Archdeacon et al. [8] In many cases, a plate has to be placed on the quadrilateral surface. However, the quadrilateral surface is adjacent to the articular surface of the acetabulum which makes it possible that a screw here penetrates into the acetabular articular cavity. Therefore, identification of danger zones where screws are at high risk of penetrating into the articular surface is essential for the success of this procedure.

Previously, several studies have attempted to evaluate the safe zones on the quadrilateral surface for acetabular screw fixation. Although a number of studies have reported safe zones for screw fixation on the posterior acetabular wall and anterior and posterior acetabular column [9–13], the safe zone on the quadrilateral surface has not been strictly defined, except the safe zone for screw fixation in the inner wall of the pelvis during the Stoppa and anterior approaches [14, 15]. 2D CT images were utilized in these studies, which was hard to exactly define and measure the safe zone under a standard condition, especially some specific ones. So, these results, obtained from 2D CT data which can easily be influenced by body position, cannot meet clinical demands entirely. Therefore, further studies are needed to get more precise definition of the “safe zone” that can easily be identified during operation. Alternatively, 3D model, transforming the scanned data into standard coordinates, could make the result more precise. Besides, due to the limited exposure of the Stoppa approach, the screw direction would be confined. In particular, when screw insertion is perpendicular to the quadrilateral surface, it is very difficult to operate with the Stoppa approach. So, incision-based research is needed.

In this study, a 3D acetabular model was “shelled” in different amplified sizes. “Shell” refers to computer terms, similar to polygons amplified and offset, but it creates an additional polygon surface that is a given distance from the original. In order to be more practical intraoperatively, it is necessary to combine the incision with precise measurement between the edge of the “safe zone”, “dangerous zone,” and some specific anatomic landmarks.

The purpose of this study is to identify the “dangerous zone” quickly and precisely during a surgery, and try to provide references for safe screw placement and a new way for the studies on the “safe zone.”

## 2. Materials and Methods

### 2.1. Data Collection

Eighty-four human subjects (38 men, 46 women with the mean age of 59.2 years. range: 16−89 years) admitted to our institution from March 2006 to August 2009 with proximal femoral fracture without pelvic and acetabular injury were recruited in this study. All patients underwent a sixteen-line pelvic helical computed tomography scan (GE, US) with 1.5 mm slices at 0.1-s intervals for imaging of the acetabulum. The raw data obtained were stored in.dicom format.

### 2.2. Model Reconstruction

**Figure 1:**
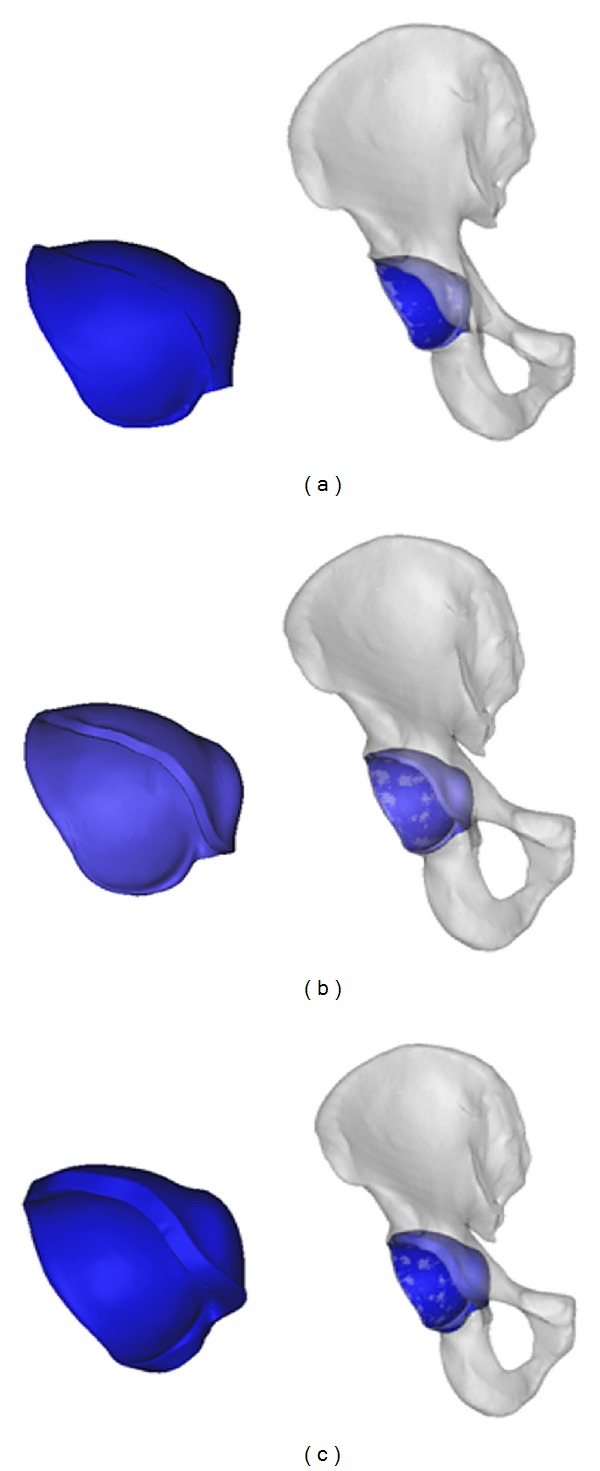
The acetabular model was stimulated from the pelvic model and “shelled” for 2.95 mm and 6 mm, respectively. (a) The acetabular model was extracted from the pelvic model; (b) the acetabular model was “shelled” for 2.95 mm and assembled with pelvic model; (c) the acetabular model was “shelled” for 6 mm and assembled with pelvic model.

3D pelvic models were reconstructed from the raw data using Mimics 12.11 software (Materialise, Leuven, Belgium), and the model of the acetabular surface was also reconstructed using Geomagic Studio 11.0 software (Geomagic, US) and HyperMesh 10.0 software (Altair, US). Two 3D acetabular models of different sizes were obtained by “shell” the original acetabular surface model for 2.95 mm and 6 mm, and then stored in STL format. The 2.95 mm was chosen as the minimum value because it is the thickness of the subchondral bone plus the radius of the typical surgical screw (the thickness of the acetabular subchondral bone which cannot be impinged during surgery [16] ranged from 0.5 mm to 1.2 mm [17–19]). Along with the minimal radius of common screws (1.75 mm), the value of 2.95 mm was obtained, while 6 mm allows for an error range of 3 mm for inserting the screw (Figure 1).

### 2.3. Surgical Incision Simulation

On the reconstructed 3D skin model, a longitudinal surgical incision along the middle of the body for the modified Stoppa approach was simulated and separated on both sides, using the methods reported in previous publications and intraoperative measurement [4].

### 2.4. Definition of the Dangerous and Safe Zone

**Figure 2:**
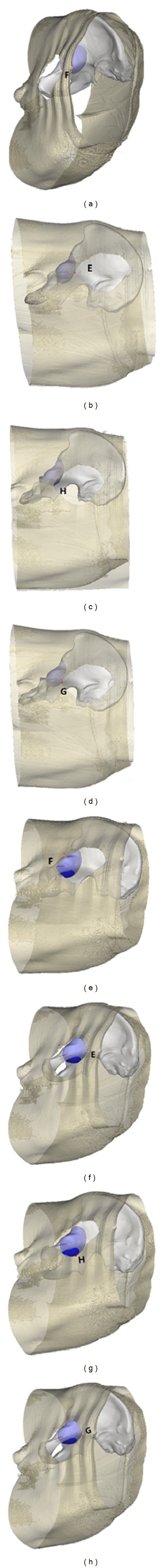
The intersection point between the acetabular model and quadrilateral surface observed from the different directions of the Stoppa incision. (a)–(d) The intersection point between “shelled” for 2.95 mm acetabular model and pelvic model, the major area of acetabular model is covered by skin, and the region obtained within the trajectory of the intersection was defined as “dangerous zone.” (e)–(h) The intersection point between “shelled” for 6 mm acetabular model and pelvic model, the major area is included in the surgical sight, and the region outside the trajectory of the intersection was defined as “safe zone”.

After the partial hyalinization of the pelvic and acetabular models, the spatial relations between these two models became visualized from the incision. The overlap area of the acetabular model “shelled” for 2.95 mm and 6 mm and the quadrilateral surface were observed at a random surgical vision from the Stoppa incision. The point of intersection between edge of skin and margin of acetabular model means that the screw inserting from this direction was along the acetabular bone tangent. The point of intersection between the acetabular model and the distal pelvic brim nearest to the obturator canal when observed from the proximal end of the Stoppa incision was designated as point F. The point of intersection between the acetabular model and the proximal pelvic brim farthest from the obturator canal when observed from the distal end of the Stoppa incision was assigned as point E. The point farthest from the obturator canal at any sight from the opposite of incision on the quadrilateral surface was assigned as point G, and the farthest point from the pelvic brim as point H (Figure 2). The observed trajectory of the acetabular model through the incision edge was designated as the safe and dangerous zone with the Stoppa approach.

### 2.5. Measurements of Safe and Dangerous Zone

**Figure 3:**
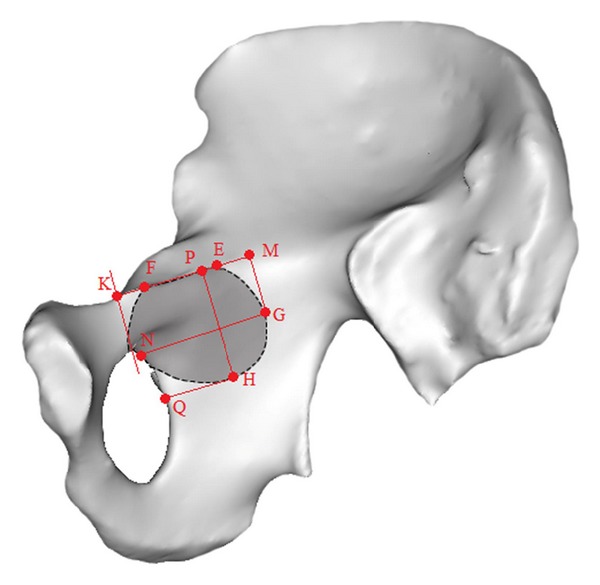
Measurements of safe and dangerous zone, the line segments EK, FK, GM, GN, HP and HQ were measured precisely.

A line perpendicular to the pelvic brim was made through the top point of the obturator foramen that intersected with the pelvic brim at point K. Two lines, respectively, parallel and perpendicular to the pelvic brim were made through point G and intersected with the pelvic brim and obturator foramen at points M and N. Point N corresponds to obturator canal where the obturator vessels and nerves cluster enter the obturator foramen. Two lines, respectively, parallel and perpendicular to the pelvic brim were made through point H and intersected with the upper edge of the pelvic brim and obturator foramen edge at points P and Q. The line segments EK, FK, GM, GN, HP, and HQ were measured (Figure 3).

## 3. Results

The reconstructed pelvic models and the assemblies with the acetabular models were shown in Figure 3.

**Figure 4:**
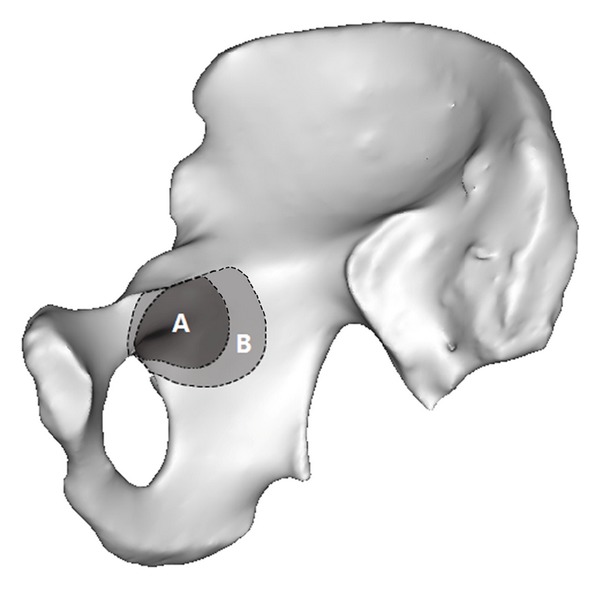
The “dangerous zone” for screw placement with the Stoppa approach. In the figure, region A represents the “dangerous zone” for screw placement when the acetabulum was amplified for 2.95 mm, which is also defined as the absolute “dangerous zone.” Region B represents the “dangerous zone” when the acetabulum was amplified for 6 mm. The part of region B outside region A is the relative “dangerous zone” and the region outside B is the absolute “safe zone.”

**Figure 5:**
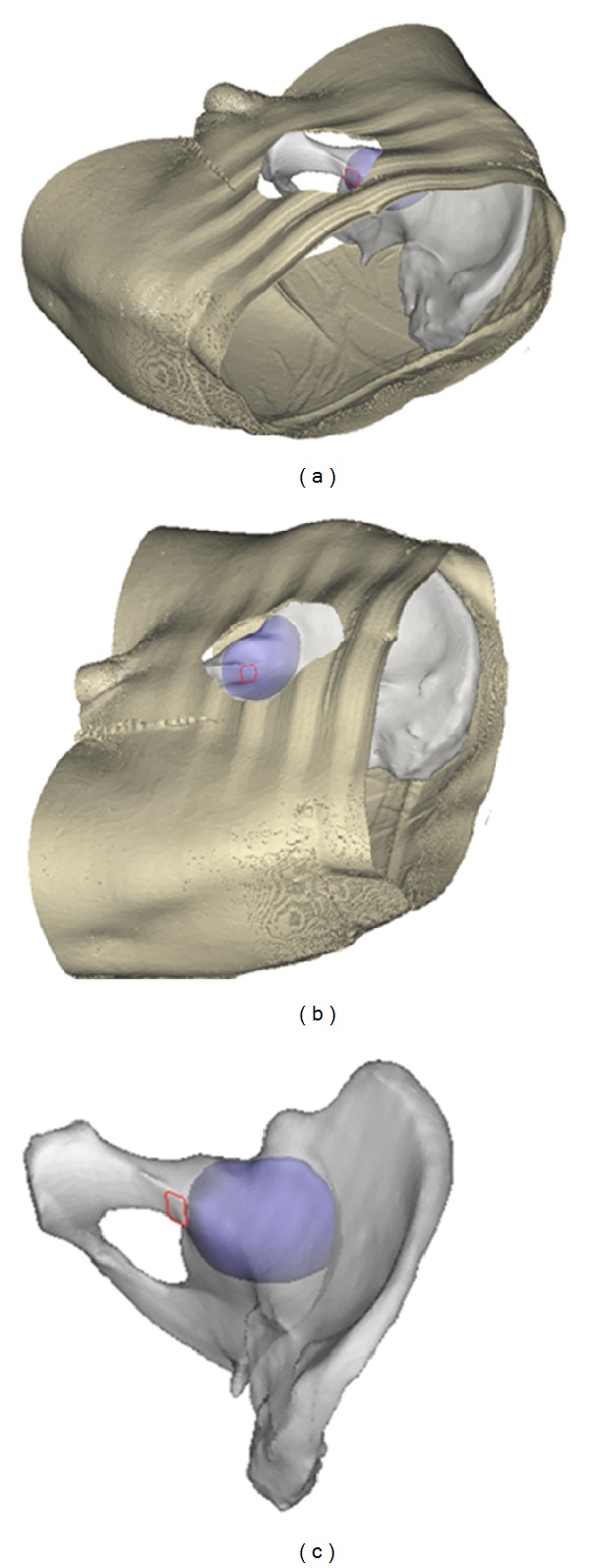
The region within the red rectangle is dangerous for screw placement with the Stoppa approach, but it becomes safe if the direction is proximal to Stoppa approach. (a) Proximal view from the Stoppa incision; (b) opposite view from the Stoppa incision; (c) the view direction proximal to Stoppa approach.

When the skin was cut and separated according to the Stoppa incision, the dangerous zone for screw placement was as shown in Figure 5. From these measurements, the length and range of the specified points during the Stoppa approach were obtained (Table 1). From Table 1 and Figure 5, it can be seen that points E, F, G, and H were important landmarks to guide screw placement during the Stoppa approach. The area from point E on the pelvic brim to point F was the “dangerous zone;” the area farther than point G to the obturator canal was within the “safe zone.” Likewise, points below H were within the “safe zone.” Region A and region B represent the danger zones when the acetabulum was “shelled” for 2.95 mm and 6 mm, respectively. The region A defined as absolute “dangerous zone” was from the pelvic brim to 3.07 cm below it and within 2.86 cm of the obturator canal, while the area outside the region B that ranges from 3.56 cm below the pelvic brim to 3.85 cm away from the obturator canal was the absolute “safe zone” for screw placement. The region within region B but outside of region A was the relatively “dangerous zone” (Figure 4).

## 4. Discussion

During the acetabular and pelvic surgery, euthyphoria of the joint is impossible. The problem of identifying the “safe zone” for the placement of screws had been a subject of debate for a long time because it was difficult to confirm whether the screw was inserted into the joint. The definition of “safe zone” for screw fixation within this area is necessary and has become popular to surgeons. Guy et al. reported the safety zone for the internal fixation of screws on the quadrilateral surface with the Stoppa [15] and anterior [14] approaches by anthropometric parameters using data from patients' CT images, pioneering similar studies. It overcomes the limited number of available cadavers and greatly contributing to the popularity of the Stoppa approach. However, it is quite difficult to conduct accurate measurements of the pelvis in a standardized body position using a 2D methodology. This safety zone can be defined more accurately using 3D reconstruction of CT images as it is less influenced by body position [20]. For the purpose of accuracy and clinical applicability, we used a 3D method in this study to evaluate the safe and “dangerous zones” on the quadrilateral surface for screw placement during surgery with the Stoppa approach.

The acetabulum is not the accurate hemisphere, and the relative correlation between the acetabulum and pelvis is hard to depict. So, proportional enlargement (shelled) of a 3D model of the acetabulum was used to preserve the anatomical relationship between them. In this study, both the “safe zone” on the quadrilateral surface (determined by measuring the spatial distance between several relatively fixed anatomic landmarks like the pelvic brim and obturator canal) and the “dangerous zone” can be identified quickly during the operation. It is easier to expose these anatomic landmarks than the ischial spine and midsciatic notch under the Stoppa approach. In addition, the point N where the obturator vessels and nerves cluster enter the obturator foramen is routinely exposed during the Stoppa approach and is relatively constant anatomically. The most common location for the plate placement (along the pelvic brim) can be determined using these landmarks to quickly identify the “safe zone.”

As the direction of screw can be seriously affected by incision during fixation, many directions are hard to achieve. As a result, the incision should be taken into consideration. For example, the zone above the obturator canal is usually considered to be the absolute “dangerous zone” during the Stoppa approach. But if the screw is inserted from a more proximal direction to Stoppa incision, this zone would be relative safe (Figure 5). Although it is impossible, apparently, the safe and dangerous zone may be changed according to different approaches. In this study, the region 3.85 cm away from the obturator canal was defined as absolute “safe zone,” which was equal to the partial result of Guy's study [14]. However, the region 3.56 cm below the pelvic brim was defined as “safe zone” in this study, but as “dangerous zone” in Guy's study. This difference may be explained by the influence of whether incision is taking into consideration.

The most common direction of screw fixation is from the opposite side of the injured pelvis when the Stoppa approach was used. After partial hyalinization of the pelvic and acetabular model, the “shelled” acetabular model became visible. When combined with Stoppa approach, the overlap areas which can be seen on the quadrilateral surface represent the dangerous area for screw placement. The edge of the dangerous area from different directions of the approach assemble is the margin of “dangerous zone.” Because the acetabulum is relative distal to the quadrilateral surface, in most cases, insertion of a screw is from the distal part of the incision except for the screw placed on the superior ramus of pubis. For example, in order to avoid accidental penetration into the joint, theoretically, if the screw is inserted from the distal end of the incision, entry point should be proximal to point E. Likewise, from the proximal end of the incision, entry point should be distal to point F. In conclusion, the “dangerous zone” was constituted as the region observed at a random visual point from the incision that the screw has the risk of penetrating into the joint. According to Table 1, the region from the pelvic brim to 3.07 cm below it and within 2.86 cm of the obturator canal was the approximate absolute “dangerous zone” (corresponding to region A in Figure 4). Conversely, the region 3.56 cm below the pelvic brim or 3.85 cm away from the obturator canal was the absolute “safe zone” (corresponding to the area outside region B in Figure 4). These regions can ease the intraoperative identification of the safe zone for screw placement.

## 5. Conclusions

The 3D method used in this study had a higher spatial resolution and greater accuracy than previous efforts. At the same time, the definition of the “safe zone” and the “dangerous zone” for screw placement in combination with the Stoppa incision made it more practical to guide the surgery. These results will greatly aid in the quick determination of optimal insertion sites during surgery in combination with the accurate measurement.

## Figures and Tables

**Table 1:** Length and range of the specified points during the Stoppa approach (cm).

Model	EK	FK	GM	GN	HP	HQ
2.95 mm	2.90 (2.58–3.22)	1.74 (1.42–2.01)	1.93 (1.53–2.36)	2.57 (2.29–2.86)	2.76 (2.46–3.07)	1.79 (1.29–2.19)
6 mm	3.47 (3.09–3.84)	1.01 (0.64–1.25)	2.37 (1.97–2.78)	3.49 (3.15–3.85)	3.34 (3.09–3.56)	2.34 (1.95–2.65)
